# Supplementary material for: The Combined Effect of Polygenic Risk Score and Prostate Health Index in Chinese Men Undergoing Prostate Biopsy
Source: J Clin Med. 2023 Feb 8;12(4):1343. doi: 10.3390/jcm12041343 (PMC9960699; doi:10.3390/jcm12041343)
Supplement: Supplementary file 1 [file jcm-12-01343-s001.zip › jcm-1974683-supplementary.pdf]

Supplementary materials

Table S1. Final 102 SNPs used in East Asian specific PRS.

SNP	CHR	Position	Risk allele	Ref allele	OR (East Asian association)	P value	Status	PMID
rs2134688	1	150772613	A	G	1.09	3.88E-04	Proxy	33398198
rs10127983	1	153923276	T	C	1.07	6.50E-04	AVL	33398198
rs56103503	1	154980351	T	C	1.06	4.92E-02	AVL	33398198
rs80237341	1	157119915	C	G	1.17	2.37E-07	AVL	33398198
rs6660538	1	163295678	A	C	1.07	6.63E-04	AVL	33398198
rs4075646	1	167135941	T	A	1.07	2.04E-04	AVL	33398198
rs613185	1	179897070	G	A	1.10	2.70E-05	Proxy	33398198
rs4245739	1	204518842	A	C	1.14	4.90E-03	AVL	33398198
rs708723	1	205739266	C	T	1.07	1.78E-04	AVL	33398198
rs9287719	2	10781975	C	T	1.05	6.37E-03	Proxy	33398198
rs9306894	2	20878105	G	A	1.13	1.15E-10	AVL	33398198
rs7591218	2	43637998	A	G	1.13	3.54E-09	AVL	33398198
rs13396048	2	62752975	G	A	1.15	5.01E-12	Proxy	33398198
rs2028900	2	85767735	C	T	1.09	5.32E-06	AVL	33398198
rs13419301	2	121103598	C	T	1.11	5.78E-09	Proxy	33398198
rs67560433	2	121373466	C	T	1.05	1.64E-02	Proxy	33398198
rs16854905	2	169012955	C	T	1.08	5.07E-04	AVL	33398198
rs77167534	2	173319930	C	T	1.17	1.04E-10	AVL	33398198
rs6743068	2	202126615	G	A	1.06	8.31E-03	Proxy	33398198
rs2292884	2	238443226	G	A	1.07	7.72E-04	AVL	33398198
rs2074840	2	242141719	C	T	1.06	3.78E-02	AVL	33398198
rs13385191	2	20888265	G	A	1.15	7.50E-08	N/C	20676098
rs7622860	3	18738940	A	C	1.08	3.55E-05	Proxy	33398198
rs7618603	3	23153062	A	C	1.10	7.49E-04	AVL	33398198
rs143745027	3	87144017	G	A	1.20	3.00E-13	AVL	33398198
rs7628934	3	87175984	C	T	1.10	8.82E-07	AVL	33398198
rs1283104	3	106962521	G	C	1.04	2.67E-02	AVL	33398198
rs1567780	3	113300183	T	C	1.09	7.97E-04	Proxy	33398198
rs2811476	3	127898501	C	A	1.14	4.86E-07	AVL	33398198
rs35006112	3	128213994	G	A	1.13	4.05E-11	AVL	33398198
rs201683	3	137562823	G	T	1.06	5.39E-03	Proxy	33398198
rs7650602	3	141147414	C	T	1.08	1.49E-04	AVL	33398198
rs2293607	3	169482335	T	C	1.05	8.43E-03	AVL	33398198
rs2055109	3	87467332	C	T	1.21	3.94E-08	N/C	22366784
rs73862213	3	128217499	G	A	1.31	5.87E-23	AVL	31562322
rs13122786	4	95544718	C	T	1.06	3.21E-03	Proxy	33398198
rs7679673	4	106061534	C	A	1.07	2.02E-03	AVL	33398198
rs17035310	4	106064754	C	T	1.18	4.68E-03	AVL	33398198
rs77821238	4	140948835	C	T	1.11	9.80E-03	AVL	33398198
rs147762399	4	152030340	T	C	1.07	1.37E-03	AVL	33398198
rs2242652	5	1280028	G	A	1.16	6.83E-15	AVL	33398198
rs2672843	5	177891551	G	A	1.08	1.15E-02	AVL	33398198
rs12653946	5	1895829	T	C	1.26	3.90E-18	AVL	20676098
rs7773037	6	21330689	C	T	1.10	4.72E-07	Proxy	33398198
rs9469899	6	34793124	A	G	1.06	8.48E-03	AVL	33398198
rs4714485	6	41536587	G	T	1.15	2.62E-12	AVL	33398198
rs6453845	6	76495882	T	G	1.09	3.81E-05	Proxy	33398198
rs339351	6	117200434	C	A	1.22	8.13E-23	AVL	33398198
rs13215045	6	153447516	C	T	1.07	6.22E-04	AVL	33398198
rs963800	6	160150279	C	T	1.09	6.38E-06	AVL	33398198
rs1983891	6	41536427	T	C	1.15	7.60E-08	N/C	20676098
rs339331	6	117210052	T	C	1.22	1.60E-12	N/C	20676098
rs12155172	7	20999211	A	G	1.07	1.82E-03	Proxy	33398198
rs6956484	7	27564862	A	C	1.14	3.80E-04	AVL	33398198
rs10486567	7	27976563	G	A	1.10	1.13E-03	AVL	33398198
rs12701838	7	40877473	A	G	1.21	1.82E-03	AVL	33398198
rs869170	7	47451918	G	A	1.04	3.46E-02	Proxy	33398198
rs6955627	7	92577760	C	T	1.07	7.68E-04	AVL	33398198
rs870167	8	8498803	G	A	1.07	1.87E-03	AVL	33398198
rs11135749	8	23470785	T	G	1.11	1.98E-03	Proxy	33398198
rs1160267	8	23529521	G	A	1.27	2.10E-40	AVL	33398198

rs12677206	8	26063165	A	C	1.10	2.64E-06	AVL	33398198
rs7463326	8	128027954	G	A	1.27	4.19E-25	AVL	33398198
rs72725879	8	128103969	T	C	1.71	9.48E-29	AVL	33398198
rs62516012	8	128342866	C	G	1.18	4.10E-04	Proxy	33398198
rs6983267	8	128413305	G	T	1.15	2.91E-13	AVL	33398198
rs10090154	8	128532137	T	C	1.61	3.88E-06	AVL	33398198
rs817872	9	110144887	C	T	1.07	4.39E-04	N/C	33398198
rs7047271	9	132573536	T	C	1.08	1.46E-04	Proxy	33398198
rs817826	9	110156300	C	T	1.41	5.45E-14	AVL	23023329
rs10993994	10	51549496	T	C	1.18	1.34E-18	AVL	33398198
rs11817544	10	80236999	C	A	1.17	2.21E-12	AVL	33398198
rs12412705	10	80835998	C	T	1.11	7.44E-10	N/C	33398198
rs1935581	10	90195149	C	T	1.05	6.45E-03	AVL	33398198
rs12262998	10	104428716	C	T	1.08	2.19E-02	AVL	33398198
rs4558107	10	122794926	A	G	1.08	2.19E-03	AVL	33398198
rs140783917	10	122834482	C	T	1.22	4.30E-14	AVL	33398198
rs2252004	10	122844709	G	T	1.16	1.98E-08	N/C	22366784
rs4554825	10	80244623	C	T	1.14	8.43E-12	N/C	31562322
rs11002805	10	80825897	A	G	1.16	5.28E-20	Proxy	31562322
rs61890184	11	7547587	A	G	1.15	7.50E-07	AVL	33398198
rs1048374	11	58902679	G	A	1.15	6.22E-09	N/C	33398198
rs17450692	11	76267331	C	T	1.08	3.45E-03	Proxy	33398198
rs878987	11	134266372	G	A	1.11	4.21E-02	AVL	33398198
rs12791447	11	7556577	C	T	1.23	3.59E-08	N/C	26443449
rs7968403	12	65012824	T	C	1.08	1.58E-03	AVL	33398198
rs67026445	12	90156377	T	C	1.12	1.55E-06	Proxy	33398198
rs77121786	12	102446675	G	T	1.06	1.28E-02	AVL	33398198
rs1270884	12	114685571	A	G	1.07	8.21E-03	AVL	33398198
rs1327653	13	51076440	T	C	1.10	2.17E-04	AVL	33398198
rs9600079	13	73728139	T	G	1.18	2.80E-09	AVL	20676098
rs12882923	14	37136194	G	A	1.10	1.63E-05	Proxy	33398198
rs8006682	14	69134264	C	G	1.07	1.11E-02	Proxy	33398198
rs58262369	14	64693912	C	T	1.28	3.05E-10	AVL	26443449
rs11561564	15	40965044	G	A	1.06	8.14E-03	AVL	33398198
rs730708	15	66942093	T	C	1.11	2.81E-11	proxy	31562322
rs410157	17	618965	T	C	1.07	1.69E-04	Proxy	33398198
rs3110641	17	36047417	A	G	1.08	2.66E-05	AVL	33398198
rs11649743	17	36074979	G	A	1.15	9.14E-13	AVL	33398198
rs11651052	17	36103565	G	A	1.31	9.68E-39	Proxy	33398198
rs8089411	18	51771322	C	T	1.07	3.10E-03	AVL	33398198
rs11876000	18	73035513	T	G	1.04	4.10E-02	AVL	33398198
rs4802297	19	38738130	G	C	1.06	3.47E-03	AVL	33398198
rs2659051	19	51345568	G	C	1.14	3.39E-13	AVL	33398198
rs103294	19	54797848	C	T	1.28	5.34E-16	AVL	23023329
rs73909841	20	49548807	T	C	1.20	9.21E-06	AVL	33398198
rs3787099	20	62307517	A	G	1.16	8.87E-03	AVL	33398198
rs5759167	22	43500212	G	T	1.09	1.09E-05	AVL	33398198
rs138708	22	39138332	G	A	1.20	1.13E-15	AVL	31562322
rs960417	X	9811095	A	G	1.04	4.51E-02	AVL	33398198
rs5972255	X	30896320	T	C	1.04	2.64E-02	AVL	33398198
rs4826594	X	54454406	A	G	1.07	7.13E-09	AVL	31562322

Abbreviation: CHR, chromosome; Ref. Reference.

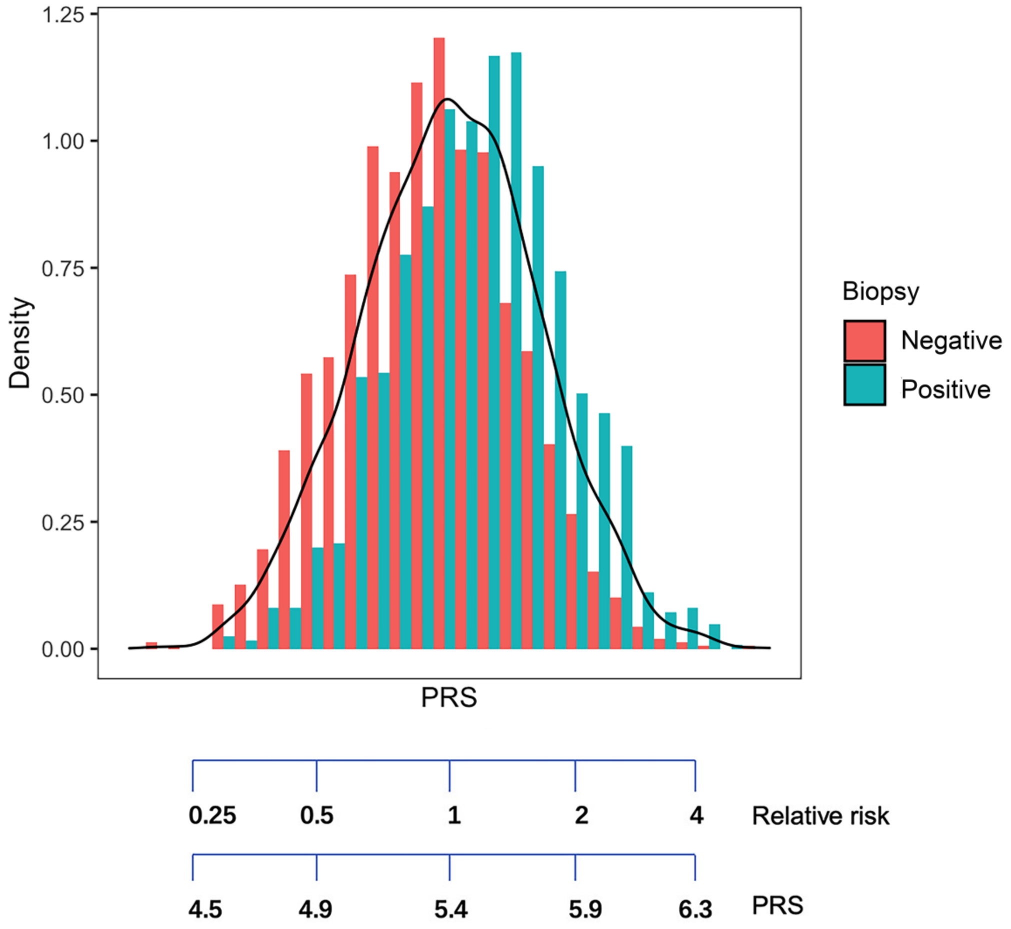

Figure S1. The PRS distribution in patients undergoing prostate biopsy.

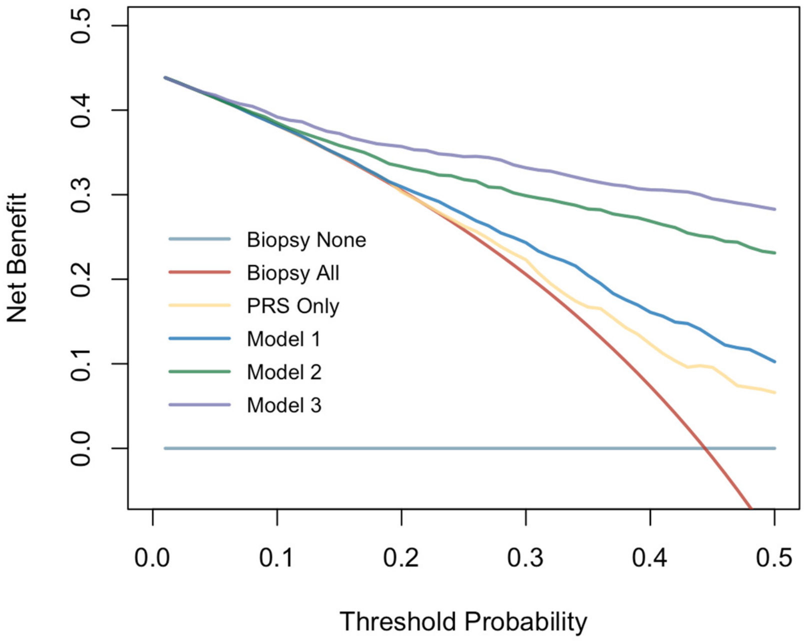

Figure S2. DCA curves of three different clinical risk model with PRS. *Model 1: Age + FH; Model 2: Age + FH + log(PV) + log(PSA) +log(%fPSA); Model3: Age + FH + log(PV) + log(phi).

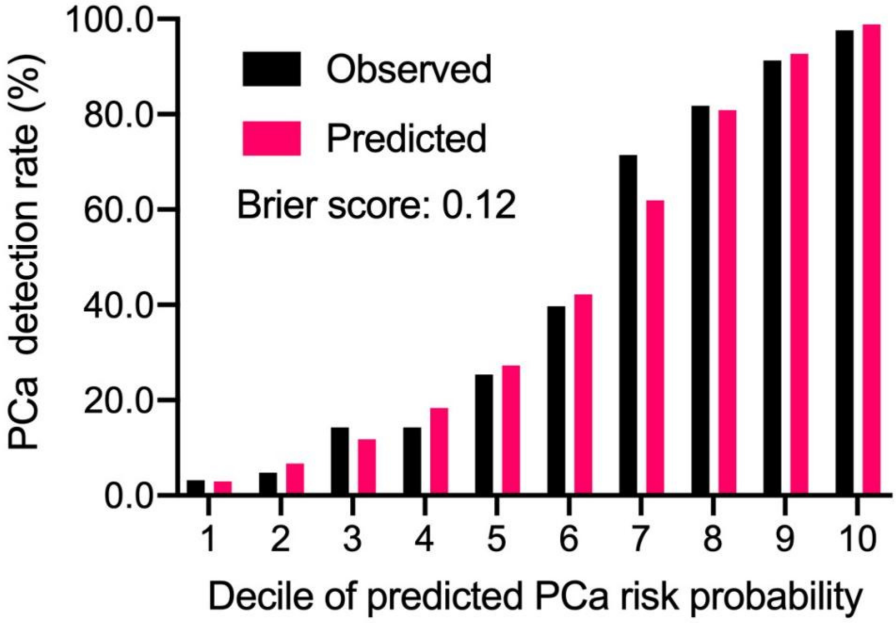

Figure S3. Calibration histogram of ***phi***-mediated model.
